# Emerging Trends in Molecular Interactions between Plants and the Broad Host Range Fungal Pathogens *Botrytis cinerea* and *Sclerotinia sclerotiorum*

**DOI:** 10.3389/fpls.2016.00422

**Published:** 2016-03-31

**Authors:** Malick Mbengue, Olivier Navaud, Rémi Peyraud, Marielle Barascud, Thomas Badet, Rémy Vincent, Adelin Barbacci, Sylvain Raffaele

**Affiliations:** LIPM, Université de Toulouse, INRA, CNRSCastanet-Tolosan, France

**Keywords:** fungal pathogen, necrotroph, broad host range, quantitative disease resistance, virulence

## Abstract

Fungal plant pathogens are major threats to food security worldwide. *Sclerotinia sclerotiorum* and *Botrytis cinerea* are closely related Ascomycete plant pathogens causing mold diseases on hundreds of plant species. There is no genetic source of complete plant resistance to these broad host range pathogens known to date. Instead, natural plant populations show a continuum of resistance levels controlled by multiple genes, a phenotype designated as quantitative disease resistance. Little is known about the molecular mechanisms controlling the interaction between plants and *S. sclerotiorum* and *B. cinerea* but significant advances were made on this topic in the last years. This minireview highlights a selection of nine themes that emerged in recent research reports on the molecular bases of plant-*S. sclerotiorum* and plant-*B. cinerea* interactions. On the fungal side, this includes progress on understanding the role of oxalic acid, on the study of fungal small secreted proteins. Next, we discuss the exchanges of small RNA between organisms and the control of cell death in plant and fungi during pathogenic interactions. Finally on the plant side, we highlight defense priming by mechanical signals, the characterization of plant Receptor-like proteins and the hormone abscisic acid in the response to *B. cinerea* and *S. sclerotiorum*, the role of plant general transcription machinery and plant small bioactive peptides. These represent nine trends we selected as remarkable in our understanding of fungal molecules causing disease and plant mechanisms associated with disease resistance to two devastating broad host range fungi.

## Introduction

A majority of studies on plant interactions with fungal pathogens over the last years have focused on specialized host–pathogen interactions. For instance the powdery mildew fungus *Blumeria graminis*, the cereal rust fungi of the *Puccinia* spp., and the corn smut fungus *Ustilago maydis* are among the most studied fungal pathogens and are obligate biotrophic pathogens restricted to a single host genus (Dean et al., 2012). Such interactions only represents a fraction of plant-fungal pathogen interactions encountered in nature and a number of broad host range fungal pathogens also are major threats for food security (Barrett et al., 2009; Dean et al., 2012). Understanding how broad host range pathogens successfully infect multiple plant lineages is a major challenge in plant pathology (Dong et al., 2015).

Among Leotiomycete, the gray mold fungus *Botrytis cinerea* and the white mold fungus *Sclerotinia sclerotiorum* stand out for having a remarkably broad host range, encompassing over 200 species. Each of these pathogens causes yearly several 100 millions of US dollars crop losses worldwide (Bolton et al., 2006; Dean et al., 2012). They are considered as typical necrotrophs, secreting an arsenal of cell wall-degrading enzymes, and toxins to kill host cells and derive energy. Host plants typically exhibit quantitative disease resistance (QDR) to *B. cinerea* and *S. sclerotiorum*, leading to a reduction rather than absence of disease (Roux et al., 2014). How these broad host range fungal pathogens cause disease and what are the genetic bases of plant QDR is still poorly understood.

**FIGURE 1:**
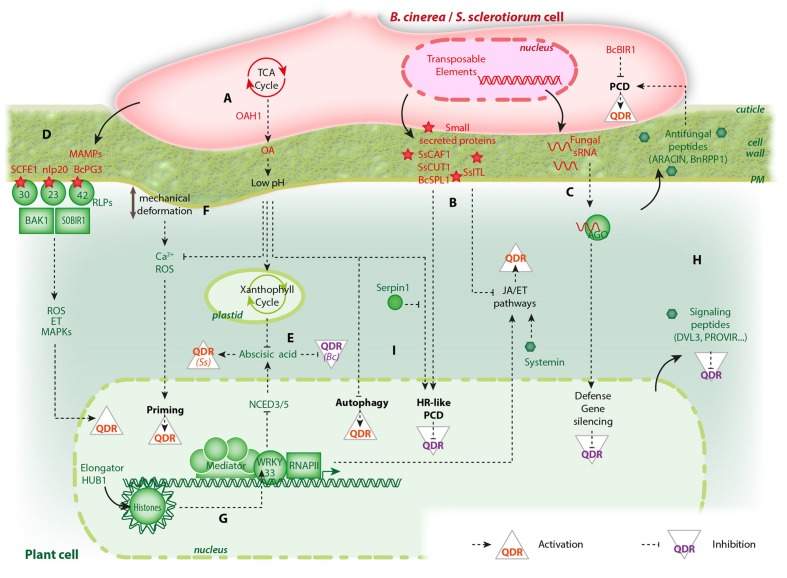
**An overview of the molecular players involved in *Botrytis cinerea*/*Sclerotinia sclerotiorum* interactions with plants and their effect on quantitative disease resistance (QDR).** Only pathways discussed in this review are shown, some elements were omitted for clarity. Fungal molecules are shown in red, plant molecules in green. **(A)** Effects of oxalic acid (OA) biosynthesis and secretion by fungi. **(B)** Small proteins secreted by fungi can activate hypersensitive response (HR)-like programmed cell death (PCD) or suppress jasmonic acid (JA) and ethylene (ET) signaling pathways to suppress QDR. **(C)** Fungal small RNAs hijack plant argonaute (AGO) proteins to suppress QDR. **(D)** Perception of microbe associated molecular patterns (MAMPs) such as SCFE1, nlp20, and BcPG3 by receptor-like proteins (RLPs) activate QDR. **(E)** The plant hormone abscisic acid can either activate QDR against *S. sclerotiorum* (Ss) or suppress QDR against *B. cinerea* (Bc). **(F)** Mechanical deformation caused by appressorium formation and fungal colonization of plant tissues prime plant cells for QDR through calcium and reactive oxygen species (ROS). **(G)** The plant general transcription machinery involves multiprotein complexes such as Elongator and Mediator that recruit the RNA polymerase II (RNAPII) to modulate gene expression upon fungal challenge. Some histone post-translational modifications are epigenetic marks altered after inoculation that regulate the activity of plant general transcription factors and control QDR. **(H)** Plant small bioactive peptides (green hexagons) have contrasted effects on QDR. **(I)** PCD in plant cells can either have a positive or negative effect on QDR depending on the type of cell death program activated.

In recent years, remarkable progress has been achieved in the characterization of fungal virulence factors and the dissection of plant response mechanisms. In this minireview, we chose to report on nine advances specifically related to pathosystems involving *B. cinerea* or *S. sclerotiorum*, concerning either the molecular bases of fungal virulence or plant QDR (summarized in **Figure 1**). The nine points presented hereafter are not meant to represent a complete overview of our current knowledge, and a number of significant discoveries could not be covered in this article. We selected nine trends based on convergent findings from multiple studies and as a source of inspiration for future studies on plant interactions with broad host range fungal pathogens.

## Putting the Role of Fungal Oxalate Secretion to the Acid Test

Oxalic acid (OA) is considered as a major virulence factor in species of the *Sclerotiniaceae* (Hegedus and Rimmer, 2005; Andrew et al., 2012). This central role of OA is further supported by the association of plant OA oxidase-related enzymes with disease resistance (Foster et al., 2012; Rietz et al., 2012). The roles of OA secretion in *S. sclerotiorum* virulence are still a matter of debate and likely include the suppression of plant defenses (Cessna et al., 2000; Williams et al., 2011), the induction of plant programmed cell death (PCD; Kim et al., 2008), the deregulation of guard cells function (Guimaraes and Stotz, 2004), and calcium detoxification (Heller and Witt-Geiges, 2013). Evidences for several of these conclusions were obtained using a UV-induced OA-deficient mutant of *S. sclerotiorum* (Godoy et al., 1990). Recently, Liang et al. (2015) generated disruptive mutants in the gene encoding oxaloacetate acetylhydrolase 1 (OAH1), an enzyme mediating OA biosynthesis (Joosten et al., 2008). This *oah1* mutant was completely abolished in OA synthesis and impaired in pathogenicity. Several phenotypic differences were noted compared to the UV-induced OA-deficient mutants, prompting for a re-evaluation of OA function during host colonization (Liang et al., 2015). To this end, Xu et al. (2015) generated *oah1* deletion mutants in another *S. sclerotiorum* strain and confirmed that their virulence varied with the pH of the host tissues. The virulence phenotype of *oah1* mutants was restored by genetic complementation and partially restored by the exogenous acidification of host tissue (Liang et al., 2015; Xu et al., 2015), suggesting that low pH, rather than a specific acidic molecule, is required for *S. sclerotiorum* full virulence. Why acidification is mediated by OA and not another organic acid during *S. sclerotiorum* infection remains unclear. *B. cinerea* was proposed to rely primarily on the production of citrate and succinate during the colonization of sunflower. The production of different organic acids suggests that *S. sclerotiorum* and *B. cinerea* differ significantly in TCA cycle regulation (Billon-Grand et al., 2012). These findings call for investigations on metabolic organization in *S. sclerotiorum* and *B. cinerea*.

## The Plenty and Hazy Fungal Small Secreted Proteins

Secondary metabolite toxins and cell wall-degrading enzymes are known to contribute to virulence of necrotrophic fungi (Choquer et al., 2007; Kabbage et al., 2015), but knowledge on the repertoires and mode of action of *S. sclerotiorum* and *B. cinerea* proteins secreted during host colonization remains limited. Mutant screens for pathogenicity defects in *S. sclerotiorum* have identified the secreted putative Ca^2+^-binding protein SsCAF1 and the secreted Cu/Zn superoxide dismutase SsSOD1 (Xu and Chen, 2013; Xiao et al., 2014). The analysis of genes down-regulated in a hypovirulent *S. sclerotiorum* strain identified the secreted integrin-like gene *SsITL* as required for full virulence (Zhu et al., 2013). Genomic and transcriptomic approaches have revealed *S. sclerotiorum* cutinase *SsCUT1* (Zhang H. et al., 2014) and the secreted cyanovirin-N homolog *SsCVNH* (Lyu et al., 2015) to be associated with plant infection. Proteomic analyses of *B. cinerea* secretome and cell wall proteome led to the identification the cerato-platanin BcSPL1 required for full virulence (Espino et al., 2010; Frías et al., 2011, 2014). SsCAF1, SsCUT1, and BcSPL1 elicited cell death in plants whereas *SsITL* was proposed to suppress plant resistance pathways, illustrating the diverse activities of fungal small secreted proteins. Given that mutations in *SsCAF1*, *SsITL*, and *SsCVNH* caused morphological defects *in vitro*, it is unclear whether these genes are directly linked to host interaction. Since the release of genome sequences for *S. sclerotiorum* and *B. cinerea* (Amselem et al., 2011), bioinformatics analyses aimed at systematically identifying candidate proteins associated with virulence. Aguileta et al. (2012) identified 21 *B. cinerea* genes harboring signatures of positive selection, including 7 encoding predicted secreted proteins. Multicriterion analyses of *S. sclerotiorum* and *B. cinerea* secretomes highlighted over 400 secreted proteins including nearly 80 virulence factor candidates (Guyon et al., 2014; Heard et al., 2015). Specific patterns of amino acid usage and conformation may also serve as a filter to classify virulence factor candidates (Badet et al., 2015). Such bioinformatics approaches remain nevertheless challenging in fungi (Sperschneider et al., 2015). Functional analyses, improved genome annotation and extensive gene expression analyses should prove useful in understanding the role of small secreted proteins in *B. cinerea* and *S. sclerotiorum* virulence.

## Host and Pathogen Cell Death Control

Pathogen recognition often triggers a hypersensitive response (HR), the rapid release of antimicrobial compounds and local PCD. The HR efficiently suppresses the growth of most biotrophic pathogens while facilitating plant colonization by *B. cinerea* and *S. sclerotiorum* (Govrin and Levine, 2000). The secretion of OA and cell death-eliciting proteins by *B. cinerea* and *S. sclerotiorum* were proposed to promote virulence by inducing host PCD (Kim et al., 2008; Frías et al., 2011). The molecular mechanisms by which *B. cinerea* and *S. sclerotiorum* manipulate host PCD remain, however, elusive. The protease inhibitor AtSerpin1 limits cell death induction by OA and plant colonization by *B. cinerea* and *S. sclerotiorum* by modulating the activity of the cysteine protease RD21 (Lampl et al., 2013). In addition, *Arabidopsis* mutants impaired in autophagy, a form of PCD involving the degradation of cytoplasmic components, were more susceptible to *B. cinerea* (Lai et al., 2011). Furthermore, *S. sclerotiorum* OA-deficient mutants trigger restricted autophagic cell death, suggesting that OA may suppress autophagy in host cells (Kabbage et al., 2013). In *Arabidopsis acd5* mutant, ceramides were associated with autophagy and enhanced susceptibility to *B. cinerea*, but these two activities may be independent (Bi et al., 2014; Magnin-Robert et al., 2015). These findings indicate that certain mechanisms of host cell death may be favorable to necrotrophic fungi whereas others would be detrimental (Dickman and de Figueiredo, 2013). The control of PCD in fungal cells also plays a crucial role in pathogenicity. Massive fungal PCD was observed at the early stages of *B. cinerea* infection, and the anti-apoptotic protein BcBIR1 was found to positively contribute to *B. cinerea* virulence. These findings suggest that fungal PCD could be triggered by plant defense molecules, and that fungal anti-apoptotic machinery is required to prevent it (Shlezinger et al., 2011). This places the control of host and fungal cell death programs at the center of the arms race taking place during plant interactions with necrotrophic fungi.

## Two-Ways Gene Silencing by Small RNAs

In eukaryotic cells, small RNAs (sRNAs) regulate a large number of biological processes, from development to immunity and pathogen virulence. These sRNAs trigger multiple RNA interference (RNAi) pathways ultimately leading to gene silencing. Using *Arabidopsis* and tomato plants infected by *B. cinerea*, Weiberg et al. (2013) showed that fungal sRNAs silence specific host immunity genes. Mutants of plant and fungal RNAi components showed reduced silencing of host immunity genes and reduced disease symptoms, respectively, (Weiberg et al., 2013). Together these results support the action of sRNAs across the species and kingdom barrier (cross-kingdom RNAi) in plant–fungi interactions, mediated by a yet unclear transfer mechanism (Sarkies and Miska, 2014; Weiberg et al., 2014, 2015). Several of these *B. cinerea* sRNA effectors originate from loci within *Boty* transposable elements (TEs), a family of mobile genetic elements associated with virulence in *B. cinerea* natural populations (Martinez et al., 2005; Weiberg et al., 2015). This suggests that TEs may contribute to the rapid evolution of sRNA effectors, similar to what has been observed for filamentous pathogen protein effectors (Raffaele et al., 2010; Raffaele and Kamoun, 2012). The genome of *S. sclerotiorum* experienced recent expansion of TEs (Amselem et al., 2011) and sRNA-producing loci have been experimentally identified in this fungus (Zhou et al., 2012). Nevertheless, whether *S. sclerotiorum* uses sRNAs as effectors and the extent to which they contribute to pathogenicity await analysis. Cross-kingdom RNAi has also been exploited to generate transgenic plants producing sRNAs that trigger fungal genes silencing (Nowara et al., 2010; Nunes and Dean, 2012; Koch et al., 2013). This strategy of host-induced gene silencing (HIGS) has been tested in model plants and crops to silence genes of various fungal and oomycete species, providing a promising approach to control diseases and study gene function in non-transformable pathogen species (Koch and Kogel, 2014; Yin et al., 2014). Whether HIGS occurs in natural plant-fungus interactions and whether it would provide an efficient way to control *B. cinerea* and *S. sclerotiorum* has not been reported yet.

## Defense Priming by Mechanical Signals

Defense priming consists in establishing a physiological state in which plants are able to mount defense responses more rapidly or more efficiently (Conrath et al., 2006). Priming follows the perception of chemical and molecular signals linked to the presence of microbes interacting with plant cells, and also the perception of physical cues (Conrath, 2011). Plant cells can also perceive strains (mechanical deformation) caused by mechanical loads (Moulia et al., 2015). During their interaction with plants, and prior to plant tissue penetration or degradation, fungal pathogens develop important mechanical loads susceptible to emit mechanical signals (MS) and prime plant defense. Mechanical loads are due to the tremendous turgor pressure (up to 8 MPa) created by water in the vacuole of appressoria and fungal cell wall mechanical properties. This mechanical stress is generally sufficient to penetrate plant cells (Tariq and Jeffries, 1984; Bastmeyer et al., 2002; Sanati Nezhad and Geitmann, 2013). Mechanosensing occurs at the plant cell level and relies on the internal mechanical state of the cell (Coutand, 2010; Hamant, 2013; Monshausen and Haswell, 2013). Mechanosensing is involved in many plant core functions including seed development, morphogenesis, gravitropism, proprioception, and interaction with symbiotic microbes (Boudaoud, 2010; Hamant and Traas, 2010; Bastien et al., 2013; Jayaraman et al., 2014; Creff et al., 2015; Landrein et al., 2015). Recent studies demonstrated the link between mechanosensing and plant immune response to *B. cinerea* in *Arabidopsis thaliana*: plants submitted to MS exhibited higher resistance to fungal infection suggesting a priming effect operated by sterile mechanosensing (Chehab et al., 2012; Benikhlef et al., 2013). Relations between mechanosensing and immune response pathways are mediated by calcium (Chehab et al., 2011; Beneloujaephajri et al., 2013), reactive oxygen species (ROS; Chehab et al., 2012; Benikhlef et al., 2013) and are jasmonic acid (JA)-independent (Benikhlef et al., 2013). Future work should aim at addressing whether mechanosensing for fungal contact or penetration *per se*, in addition to PAMP perception, leads to enhanced plant immunity. Quantifying the MS intensity perceived by the plant will be required to this end. Quantitative biomechanical plant cell models (Barbacci et al., 2013; Ali et al., 2014) are required first milestones toward a better understanding of the molecular mechanisms underlying plant resistance priming by MS.

## Perception of Fungi by Plant Receptor Like Proteins

Detection of microbe-associated molecular patterns (MAMPs) is an important part of the plant defense against pathogens (Boller and Felix, 2009). It relies on plasma membrane resident pattern recognition receptors (PRRs) able to perceive pathogen signatures in the apoplastic space and to activate a downstream signaling through their kinase domain (Monaghan and Zipfel, 2012). In plants, early detection of fungal pathogens occurs mainly through PRRs-mediated perception of chitin (Sanchez-Vallet et al., 2015). Peptide-mediated fungal perception is also becoming increasingly documented. *S. sclerotiorum* produces a protein elicitor (SCFE1) that triggers oxidative burst, ethylene production, mitogen-activated protein kinase activation and gene induction (Zhang et al., 2013). Natural variation among *Arabidopsis* accessions and mutants identified *RLP30* as required for all SCFE1 responses. *RLP30* encodes for a PRR devoid of a kinase domain, suggesting the involvement of co-regulators for intracellular signaling. In a similar approach Albert et al. (2015) screened for *Arabidopsis* mutants and natural accessions unresponsive to nlp20, a 20 amino acid conserved peptide derived from a class of necrosis inducing proteins found in bacteria, oomycetes and fungi (Albert et al., 2015). Upon nlp20 binding, the RLP23 receptor forms a ternary complex required for signaling with two other kinase-containing co-receptors, BAK1 and SOBIR1 (Albert et al., 2015). Finally, the *Arabidopsis RLP42* gene is required for *Arabidopsis* responses to exogenous application of *B. cinerea* polygalacturonase 3 (Zhang L. et al., 2014). Given that RLP30, RLP42, and RLP23 are homologs, it is reasonable to hypothesize that all use BAK1 or SOBIR1 as co-receptors. Because SCFE1-responsive plants included *Arabidopsis* accessions with various levels of resistance, Zhang et al. (2013) proposed that redundant elicitor perception systems involve other PRRs in addition to RLP30. This is consistent with several perception and response mechanisms acting simultaneously to trigger QDR (Roux et al., 2014). The discoveries of RLP23, RLP42, and soybean RLPs identified through quantitative trait loci mapping strengthen this view (Zhang L. et al., 2014; Albert et al., 2015; Zhao et al., 2015). Pyramiding several PRRs into plants would likely yield increased and long-lived resistance to devastating pathogens.

## Contrasted Impacts of Abscisic Acid on QDR

Plant hormones classically associated with resistance to necrotrophic pathogens are JA and ethylene (ET), whereas salicylic acid is associated with stimulation of resistance against biotrophic pathogens (Glazebrook, 2005). Abscisic acid (ABA) has roles in plant development and response to abiotic stress, and has also contrasted impact on plant diseases, depending notably on the pathogen infection strategy (Robert-Seilaniantz et al., 2011). ABA was shown to promote susceptibility to *B. cinerea* in tomato through alterations of the plant cuticle and C:N metabolism (Audenaert et al., 2002; Curvers et al., 2010; Seifi et al., 2013). In *A. thaliana*, Liu et al. (2015) revealed that loss of WRKY33 results in elevated ABA levels and high susceptibility to *B. cinerea*. WRKY33 limits ABA accumulation in *B. cinerea*-challenged plants by binding to the ABA biosynthesis genes *NCED3* and *NCED5* to suppress their expression. Consistently, the transmembrane receptor-like kinase AtLYK3 was proposed to act as a positive regulator of late responses to ABA and negative regulator of defenses to *B. cinerea* (Paparella et al., 2014). By contrast, inoculation of *A. thaliana* mutants revealed that ABA contributes to resistance to *S. sclerotiorum*, although it remains unclear whether ABA synthesis or perception is required for resistance (Guimaraes and Stotz, 2004; Perchepied et al., 2010). OA secreted by *S. sclerotiorum* was proposed to favor infection by inhibiting ABA-mediated stomatal closure (Guimaraes and Stotz, 2004). Zhou et al. (2015) showed that pH modulation by *S. sclerotiorum* correlates with increased synthesis of photoprotective compounds of the xanthophyll cycle that serve as precursors for ABA synthesis. Depletion in ABA precursors was suggested to account for reduced ABA levels in *S. sclerotiorum*-infected leaves and plant susceptibility (Zhou et al., 2015). Differences in the dynamics of OA secretion (Billon-Grand et al., 2012) may contribute to the contrasting impact of ABA on resistance toward *S. sclerotiorum* and *B. cinerea*. Furthermore, *B. cinerea*, but not *S. sclerotiorum*, synthesizes ABA (Siewers et al., 2004). Further studies will be required to fully understand how plants and fungi interfere with ABA pathways to modulate the outcome of infection.

## Interactions with Fungi Shed Light on Host General Transcription Machinery

Host transcriptional reprogramming after pathogen challenge is paramount in the establishment of plant defense. The general transcription machinery relies on the mediator complex, a multiprotein co-activator scaffold acting as a bridge between RNA polymerase II (RNAPII) and transcription factors (Samanta and Thakur, 2015). Plants mutated in some mediator subunits show compromised resistance to both bacterial and fungal pathogens (Zhang et al., 2012). Further, the mediator complex is a target of the HaRL44 downy mildew effector (Caillaud et al., 2013), highlighting its relevance in plant defense. Mutations in MED25 or MED16 subunits abolished the induction of JA-responsive genes and reduced resistance to *B. cinerea* (Kidd et al., 2009; Zhang et al., 2012). MED16 physically associates with the plant defense regulator WRKY33 to recruit RNAPII and activate plant genes involved in JA/ET cross-talks (Wang et al., 2015b). By contrast, mutation in the CDK8 mediator subunit caused enhanced resistance to *B. cinerea* via the regulation of the biosynthesis of cuticular waxes and secondary metabolites (Zhu et al., 2014). The Elongator is another RNAP II-interacting complex required for the induction of JA/ET defense pathways and resistance to *B. cinerea*. The Elongator subunit ELP2 is required for histone acetylation and the induction of WRKY33 and defensin genes, suggesting that Elongator-mediated histone acetylation may be required for full activation of transcriptional responses to *B. cinerea* (Wang et al., 2015a,b). HUB1 encodes a RING E3 ligase that monoubiquitinates histone H2B, interacts with mediator subunit MED21, and positively controls resistance to *B. cinerea* in an ET-dependent manner (Dhawan et al., 2009). Consistently, silencing of HUB1 orthologs in tomato increased susceptibility to *B. cinerea* and downregulated JA/ET pathway genes (Zhang et al., 2015). Pathogen responses helped deciphering the function of general transcription complexes in plants but whether *B. cinerea* and *S. sclerotiorum* are able to manipulate these complexes or associated epigenetic processes is not known.

## Plant Small Bioactive Peptides

Small bioactive peptides are defined as proteins of about 100 amino acids (aa) with roles in plant development, reproduction or interaction with the environment (Tavormina et al., 2015). They can exhibit a direct antifungal activity in the extracellular space, such as the *Brassicaceae*-specific ARACIN1 and 2. These ∼80 aa peptides display antifungal activity *in vitro* (Neukermans et al., 2015). Ectopic expression of ARACIN1 in *Arabidopsis* reduced infection by *B. cinerea*. Antifungal activity against *S. sclerotiorum* and *B. cinerea* has also been shown *in vitro* for the *Brassica napus* 35 aa proline-rich peptide BnPRP1. The *BnPRP1* gene is induced upon *S. sclerotiorum* inoculation in susceptible but not in resistant plants (Cao et al., 2015). Small bioactive peptides can also serve as intra- or intercellular signals modulating plant defense signaling pathways. In tomato, systemin-mediated activation of the JA signaling pathways is required for resistance against *B. cinerea* (El Oirdi et al., 2011). Systemin is a *Solanaceae*-specific 18 aa peptide derived from the prosystemin precursor released into the vascular system at sites of cell damage (Pearce et al., 2001). Overexpression of prosystemin increased resistance to *B. cinerea* suggesting that systemin may serve as damage signal during fungal infection (Coppola et al., 2015). Dobon et al. (2015) identified four transcription factors that confer enhanced resistance to *B. cinerea* and found 77 genes up-regulated in the four corresponding mutants. Among these were several small signaling peptides (such as devil/rotundifolia peptide DVL3) and 13 small predicted secreted proteins of unknown function of 34–123 aa, named PROVIR1 to 13. Most of these small peptide genes were induced upon fungal infection and their over-expression caused enhanced susceptibility to necrotrophic fungi (Dobon et al., 2015).

## Concluding Statement

Due their phylogenetic proximity and similarities in lifestyle, *B. cinerea* and *S. sclerotiorum* are often used interchangeably as models of broad host range necrotrophic fungi. Recent progress has revealed commonalities in their virulence strategies and in the corresponding plant responses, but also differences providing valuable insights into the diversity of the molecular bases of broad of host range pathogenicity and plant QDR.

## Author Contributions

All authors listed, have made substantial, direct and intellectual contribution to the work, and approved it for publication.

## Conflict of Interest Statement

The authors declare that the research was conducted in the absence of any commercial or financial relationships that could be construed as a potential conflict of interest.

## References

[B1] Aguileta G. Lengelle J. Chiapello H. Giraud T. Viaud M. Fournier E. (2012). Genes under positive selection in a model plant pathogenic fungus, Botrytis. *Infect. Genet. Evol.* 12 987–996. 10.1016/j.meegid.2012.02.012 22406010

[B2] Albert I. Böhm H. Albert M. Feiler C. E. Imkampe J. Wallmeroth N. (2015). An RLP23–SOBIR1–BAK1 complex mediates NLP-triggered immunity. *Nat. Plants* 1 15140 10.1038/nplants.2015.140 27251392

[B3] Ali O. Mirabet V. Godin C. Traas J. (2014). Physical models of plant development. *Annu. Rev. Cell Dev. Biol.* 30 59–78. 10.1146/annurev-cellbio-101512-122410 25000996

[B4] Amselem J. Cuomo C. A. van Kan J. A. Viaud M. Benito E. P. Couloux A. (2011). Genomic analysis of the necrotrophic fungal pathogens *Sclerotinia sclerotiorum* and *Botrytis cinerea*. *PLoS Genet.* 7:e1002230 10.1371/journal.pgen.1002230 PMC3158057 21876677

[B5] Andrew M. Barua R. Short S. M. Kohn L. M. (2012). Evidence for a common toolbox based on necrotrophy in a fungal lineage spanning necrotrophs, biotrophs, endophytes, host generalists and specialists. *PLoS ONE* 7:e29943 10.1371/journal.pone.0029943 PMC3256194 22253834

[B6] Audenaert K. De Meyer G. B. Hofte M. M. (2002). Abscisic acid determines basal susceptibility of tomato to *Botrytis cinerea* and suppresses salicylic acid-dependent signaling mechanisms. *Plant Physiol.* 128 491–501. 10.1104/pp.010605 11842153 PMC148912

[B7] Badet T. Peyraud R. Raffaele S. (2015). Common protein sequence signatures associate with *Sclerotinia borealis* lifestyle and secretion in fungal pathogens of the Sclerotiniaceae. *Front. Plant Sci.* 6:776 10.3389/fpls.2015.00776 PMC4585107 26442085

[B8] Barbacci A. Lahaye M. Magnenet V. (2013). Another brick in the cell wall: biosynthesis dependent growth model. *PLoS ONE* 8:e74400 10.1371/journal.pone.0074400 PMC3774806 24066142

[B9] Barrett L. G. Thrall P. H. Dodds P. N. van der Merwe M. Linde C. C. Lawrence G. J. (2009). Diversity and evolution of effector loci in natural populations of the plant pathogen *Melampsora lini*. *Mol. Biol. Evol.* 26 2499–2513. 10.1093/molbev/msp166 19633228 PMC2767095

[B10] Bastien R. Bohr T. Moulia B. Douady S. (2013). Unifying model of shoot gravitropism reveals proprioception as a central feature of posture control in plants. *Proc. Natl. Acad. Sci. U.S.A.* 110 755–760. 10.1073/pnas.1214301109 23236182 PMC3545775

[B11] Bastmeyer M. Deising H. B. Bechinger C. (2002). Force exertion in fungal infection. *Annu. Rev. Biophys. Biomol. Struct.* 31 321–341. 10.1146/annurev.biophys.31.091701.170951 11988473

[B12] Beneloujaephajri E. Costa A. L’Haridon F. Metraux J. P. Binda M. (2013). Production of reactive oxygen species and wound-induced resistance in *Arabidopsis thaliana* against *Botrytis cinerea* are preceded and depend on a burst of calcium. *BMC Plant Biol.* 13:160 10.1186/1471-2229-13-160 PMC4016300 24134148

[B13] Benikhlef L. L’Haridon F. Abou-Mansour E. Serrano M. Binda M. Costa A. (2013). Perception of soft mechanical stress in *Arabidopsis* leaves activates disease resistance. *BMC Plant Biol.* 13:133 10.1186/1471-2229-13-133 PMC3848705 24033927

[B14] Bi F. C. Liu Z. Wu J. X. Liang H. Xi X. L. Fang C. (2014). Loss of ceramide kinase in *Arabidopsis* impairs defenses and promotes ceramide accumulation and mitochondrial H2O2 bursts. *Plant Cell* 26 3449–3467. 10.1105/tpc.114.127050 25149397 PMC4176443

[B15] Billon-Grand G. Rascle C. Droux M. Rollins J. A. Poussereau N. (2012). pH modulation differs during sunflower cotyledon colonization by the two closely related necrotrophic fungi *Botrytis cinerea* and *Sclerotinia sclerotiorum*. *Mol. Plant Pathol.* 13 568–578. 10.1111/j.1364-3703.2011.00772.x 22171786 PMC6638627

[B16] Boller T. Felix G. (2009). A renaissance of elicitors: perception of microbe-associated molecular patterns and danger signals by pattern-recognition receptors. *Annu. Rev. Plant Biol.* 60 379–406. 10.1146/annurev.arplant.57.032905.105346 19400727

[B17] Bolton M. D. Thomma B. P. H. J. Nelson B. D. (2006). *Sclerotinia sclerotiorum* (Lib.) de Bary: biology and molecular traits of a cosmopolitan pathogen. *Mol. Plant Pathol.* 7 1–16. 10.1111/j.1364-3703.2005.00316.x 20507424

[B18] Boudaoud A. (2010). An introduction to the mechanics of morphogenesis for plant biologists. *Trends Plant Sci.* 15 353–360. 10.1016/j.tplants.2010.04.002 20427223

[B19] Caillaud M. C. Asai S. Rallapalli G. Piquerez S. Fabro G. Jones J. D. (2013). A downy mildew effector attenuates salicylic acid-triggered immunity in *Arabidopsis* by interacting with the host mediator complex. *PLoS Biol.* 11:e1001732 10.1371/journal.pbio.1001732 PMC3858237 24339748

[B20] Cao H. Ke T. Liu R. Yu J. Dong C. Cheng M. (2015). Identification of a novel proline-rich antimicrobial peptide from *Brassica napus*. *PLoS ONE* 10:e0137414 10.1371/journal.pone.0137414 PMC4575134 26383098

[B21] Cessna S. G. Sears V. E. Dickman M. B. Low P. S. (2000). Oxalic acid, a pathogenicity factor for *Sclerotinia sclerotiorum*, suppresses the oxidative burst of the host plant. *Plant Cell* 12 2191–2200. 10.2307/3871114 11090218 PMC150167

[B22] Chehab E. W. Wang Y. Braam J. (2011). “Mechanical force responses of plant cells and plants,” in *Mechanical Integration of Plant Cells and Plants*, ed. Wojtaszek P. (Heidelberg: Springer-Verlag Berlin), 173–194.

[B23] Chehab E. W. Yao C. Henderson Z. Kim S. Braam J. (2012). *Arabidopsis* touch-induced morphogenesis is jasmonate mediated and protects against pests. *Curr. Biol.* 22 701–706. 10.1016/j.cub.2012.02.061 22483939

[B24] Choquer M. Fournier E. Kunz C. Levis C. Pradier J. M. Simon A. (2007). *Botrytis cinerea* virulence factors: new insights into a necrotrophic and polyphageous pathogen. *FEMS Microbiol. Lett.* 277 1–10. 10.1111/j.1574-6968.2007.00930.x 17986079

[B25] Conrath U. (2011). Molecular aspects of defence priming. *Trends Plant Sci.* 16 524–531. 10.1016/j.tplants.2011.06.004 21782492

[B26] Conrath U. Beckers G. J. Flors V. Garcia-Agustin P. Jakab G. Mauch F. (2006). Priming: getting ready for battle. *Mol. Plant Microbe Interact.* 19 1062–1071. 10.1094/MPMI-19-1062 17022170

[B27] Coppola M. Corrado G. Coppola V. Cascone P. Martinelli R. Digilio M. C. (2015). Prosystemin overexpression in tomato enhances resistance to different biotic stresses by activating genes of multiple signaling pathways. *Plant Mol. Biol. Rep.* 33 1270–1285. 10.1007/s11105-014-0834-x 26339120 PMC4551541

[B28] Coutand C. (2010). Mechanosensing and thigmomorphogenesis, a physiological and biomechanical point of view. *Plant Sci.* 179 168–182. 10.1016/j.plantsci.2010.05.001

[B29] Creff A. Brocard L. Ingram G. (2015). A mechanically sensitive cell layer regulates the physical properties of the *Arabidopsis* seed coat. *Nat. Commun.* 6 6382 10.1038/ncomms7382 25702924

[B30] Curvers K. Seifi H. Mouille G. de Rycke R. Asselbergh B. Van Hecke A. (2010). Abscisic acid deficiency causes changes in cuticle permeability and pectin composition that influence tomato resistance to *Botrytis cinerea*. *Plant Physiol.* 154 847–860. 10.1104/pp.110.158972 20709830 PMC2949027

[B31] Dean R. Van Kan J. A. L. Pretorius Z. A. Hammond-Kosack K. E. Di Pietro A. Spanu P. D. (2012). The Top 10 fungal pathogens in molecular plant pathology. *Mol. Plant Pathol.* 13 414–430. 10.1111/j.1364-3703.2011.00783.x 22471698 PMC6638784

[B32] Dhawan R. Luo H. Foerster A. M. Abuqamar S. Du H. N. Briggs S. D. (2009). HISTONE MONOUBIQUITINATION1 interacts with a subunit of the mediator complex and regulates defense against necrotrophic fungal pathogens in *Arabidopsis*. *Plant Cell* 21 1000–1019. 10.1105/tpc.108.062364 19286969 PMC2671699

[B33] Dickman M. B. de Figueiredo P. (2013). Death be not proud -cell death control in plant fungal interactions. *PLoS Pathog.* 9:e1003542 10.1371/journal.ppat.1003542 PMC3771904 24068920

[B34] Dobon A. Canet J. V. Garcia-Andrade J. Angulo C. Neumetzler L. Persson S. (2015). Novel disease susceptibility factors for fungal necrotrophic pathogens in *Arabidopsis*. *PLoS Pathog.* 11:e1004800 10.1371/journal.ppat.1004800 PMC4382300 25830627

[B35] Dong S. Raffaele S. Kamoun S. (2015). The two-speed genomes of filamentous pathogens: waltz with plants. *Curr. Opin. Genet. Dev.* 35 57–65. 10.1016/j.gde.2015.09.001 26451981

[B36] El Oirdi M. El Rahman T. A. Rigano L. El Hadrami A. Rodriguez M. C. Daayf F. (2011). *Botrytis cinerea* manipulates the antagonistic effects between immune pathways to promote disease development in tomato. *Plant Cell* 23 2405–2421. 10.1105/tpc.111.083394 21665999 PMC3160041

[B37] Espino J. J. Gutierrez-Sanchez G. Brito N. Shah P. Orlando R. Gonzalez C. (2010). The *Botrytis cinerea* early secretome. *Proteomics* 10 3020–3034. 10.1002/pmic.201000037 20564262 PMC3983782

[B38] Foster J. Kim H. U. Nakata P. A. Browse J. (2012). A previously unknown oxalyl-CoA synthetase is important for oxalate catabolism in *Arabidopsis*. *Plant Cell* 24 1217–1229. 10.1105/tpc.112.096032 22447686 PMC3336115

[B39] Frías M. Brito N. Gonzalez M. Gonzalez C. (2014). The phytotoxic activity of the cerato-platanin BcSpl1 resides in a two-peptide motif on the protein surface. *Mol. Plant Pathol.* 15 342–351. 10.1111/mpp.12097 24175916 PMC6638778

[B40] Frías M. González C. Brito N. (2011). BcSpl1, a cerato-platanin family protein, contributes to *Botrytis cinerea* virulence and elicits the hypersensitive response in the host. *New Phytol.* 192 483–495. 10.1111/j.1469-8137.2011.03802.x 21707620

[B41] Glazebrook J. (2005). Contrasting mechanisms of defense against biotrophic and necrotrophic pathogens. *Annu. Rev. Phytopathol.* 43 205–227. 10.1146/annurev.phyto.43.040204.135923 16078883

[B42] Godoy G. Steadman J. Dickman M. Dam R. (1990). Use of mutants to demonstrate the role of oxalic acid in pathogenicity of *Sclerotinia sclerotiorum* on *Phaseolus vulgaris*. *Physiol. Mol. Plant Pathol.* 37 179–191. 10.1016/0885-5765(90)90010-U

[B43] Govrin E. M. Levine A. (2000). The hypersensitive response facilitates plant infection by the necrotrophic pathogen *Botrytis cinerea*. *Curr. Biol.* 10 751–757. 10.1016/S0960-9822(00)00560-1 10898976

[B44] Guimaraes R. L. Stotz H. U. (2004). Oxalate production by *Sclerotinia sclerotiorum* deregulates guard cells during infection. *Plant Physiol.* 136 3703–3711. 10.1104/pp.104.049650 15502012 PMC527168

[B45] Guyon K. Balagué C. Roby D. Raffaele S. (2014). Secretome analysis reveals effector candidates associated with broad host range necrotrophy in the fungal plant pathogen *Sclerotinia sclerotiorum*. *BMC Genomics* 15:336 10.1186/1471-2164-15-336 PMC4039746 24886033

[B46] Hamant O. (2013). Widespread mechanosensing controls the structure behind the architecture in plants. *Curr. Opin. Plant Biol.* 16 654–660. 10.1016/j.pbi.2013.06.006 23830994

[B47] Hamant O. Traas J. (2010). The mechanics behind plant development. *New Phytol.* 185 369–385. 10.1111/j.1469-8137.2009.03100.x 20002316

[B48] Heard S. Brown N. A. Hammond-Kosack K. (2015). An interspecies comparative analysis of the predicted secretomes of the necrotrophic plant pathogens *Sclerotinia sclerotiorum* and *Botrytis cinerea*. *PLoS ONE* 10:e0130534 10.1371/journal.pone.0130534 PMC4480369 26107498

[B49] Hegedus D. D. Rimmer S. R. (2005). *Sclerotinia sclerotiorum*: when “to be or not to be” a pathogen? *FEMS Microbiol. Lett.* 251 177–184. 10.1016/j.femsle.2005.07.040 16112822

[B50] Heller A. Witt-Geiges T. (2013). Oxalic acid has an additional, detoxifying function in *Sclerotinia sclerotiorum* pathogenesis. *PLoS ONE* 8:e72292 10.1371/journal.pone.0072292 PMC3741138 23951305

[B51] Jayaraman D. Gilroy S. Ane J. M. (2014). Staying in touch: mechanical signals in plant-microbe interactions. *Curr. Opin. Plant Biol.* 20 104–109. 10.1016/j.pbi.2014.05.003 24875767

[B52] Joosten H. J. Han Y. Niu W. Vervoort J. Dunaway-Mariano D. Schaap P. J. (2008). Identification of fungal oxaloacetate hydrolyase within the isocitrate lyase/PEP mutase enzyme superfamily using a sequence marker-based method. *Proteins* 70 157–166. 10.1002/prot.21622 17654546

[B53] Kabbage M. Williams B. Dickman M. B. (2013). Cell death control: the interplay of apoptosis and autophagy in the pathogenicity of *Sclerotinia sclerotiorum*. *PLoS Pathog.* 9:e1003287 10.1371/journal.ppat.1003287 PMC3623803 23592997

[B54] Kabbage M. Yarden O. Dickman M. B. (2015). Pathogenic attributes of *Sclerotinia sclerotiorum*: switching from a biotrophic to necrotrophic lifestyle. *Plant Sci.* 233 53–60. 10.1016/j.plantsci.2014.12.018 25711813

[B55] Kidd B. N. Edgar C. I. Kumar K. K. Aitken E. A. Schenk P. M. Manners J. M. (2009). The mediator complex subunit PFT1 is a key regulator of jasmonate-dependent defense in *Arabidopsis*. *Plant Cell* 21 2237–2252. 10.1105/tpc.109.066910 19671879 PMC2751954

[B56] Kim K. S. Min J. Y. Dickman M. B. (2008). Oxalic acid is an elicitor of plant programmed cell death during *Sclerotinia sclerotiorum* disease development. *Mol. Plant Microbe Interact.* 21 605–612. 10.1094/MPMI-21-5-0605 18393620

[B57] Koch A. Kogel K. H. (2014). New wind in the sails: improving the agronomic value of crop plants through RNAi-mediated gene silencing. *Plant Biotechnol. J.* 12 821–831. 10.1111/pbi.12226 25040343

[B58] Koch A. Kumar N. Weber L. Keller H. Imani J. Kogel K. H. (2013). Host-induced gene silencing of cytochrome P450 lanosterol C14alpha-demethylase-encoding genes confers strong resistance to *Fusarium* species. *Proc. Natl. Acad. Sci. U.S.A.* 110 19324–19329. 10.1073/pnas.1306373110 24218613 PMC3845197

[B59] Lai Z. Wang F. Zheng Z. Fan B. Chen Z. (2011). A critical role of autophagy in plant resistance to necrotrophic fungal pathogens. *Plant J.* 66 953–968. 10.1111/j.1365-313X.2011.04553.x 21395886

[B60] Lampl N. Alkan N. Davydov O. Fluhr R. (2013). Set-point control of RD21 protease activity by AtSerpin1 controls cell death in *Arabidopsis*. *Plant J.* 74 498–510. 10.1111/tpj.12141 23398119

[B61] Landrein B. Kiss A. Sassi M. Chauvet A. Das P. Cortizo M. (2015). Mechanical stress contributes to the expression of the STM homeobox gene in *Arabidopsis* shoot meristems. *Elife* 4:e07811 10.7554/eLife.07811 PMC4666715 26623515

[B62] Liang X. Liberti D. Li M. Kim Y. T. Hutchens A. Wilson R. (2015). Oxaloacetate acetylhydrolase gene mutants of *Sclerotinia sclerotiorum* do not accumulate oxalic acid, but do produce limited lesions on host plants. *Mol. Plant Pathol.* 16 559–571. 10.1111/mpp.12211 25285668 PMC6638444

[B63] Liu S. Kracher B. Ziegler J. Birkenbihl R. P. Somssich I. E. (2015). Negative regulation of ABA signaling by WRKY33 is critical for *Arabidopsis* immunity towards *Botrytis cinerea* 2100. *Elife* 4:e07295 10.7554/eLife.07295 PMC4487144 26076231

[B64] Lyu X. Shen C. Fu Y. Xie J. Jiang D. Li G. (2015). Comparative genomic and transcriptional analyses of the carbohydrate-active enzymes and secretomes of phytopathogenic fungi reveal their significant roles during infection and development. *Sci. Rep.* 5 15565 10.1038/srep15565 PMC4632110 26531059

[B65] Magnin-Robert M. Le Bourse D. Markham J. Dorey S. Clement C. Baillieul F. (2015). Modifications of sphingolipid content affect tolerance to hemibiotrophic and necrotrophic pathogens by modulating plant defense responses in *Arabidopsis*. *Plant Physiol.* 169 2255–2274. 10.1104/pp.15.01126 26378098 PMC4634087

[B66] Martinez F. Dubos B. Fermaud M. (2005). The role of saprotrophy and virulence in the population dynamics of *Botrytis cinerea* in vineyards. *Phytopathology* 95 692–700. 10.1094/PHYTO-95-0692 18943786

[B67] Monaghan J. Zipfel C. (2012). Plant pattern recognition receptor complexes at the plasma membrane. *Curr. Opin. Plant Biol.* 15 349–357. 10.1016/j.pbi.2012.05.006 22705024

[B68] Monshausen G. B. Haswell E. S. (2013). A force of nature: molecular mechanisms of mechanoperception in plants. *J. Exp. Bot.* 64 4663–4680. 10.1093/jxb/ert204 23913953 PMC3817949

[B69] Moulia B. Coutand C. Julien J. L. (2015). Mechanosensitive control of plant growth: bearing the load, sensing, transducing, and responding. *Front. Plant Sci.* 6:52 10.3389/fpls.2015.00052 PMC4337334 25755656

[B70] Neukermans J. Inze A. Mathys J. De Coninck B. van de Cotte B. Cammue B. P. (2015). ARACINs, Brassicaceae-specific peptides exhibiting antifungal activities against necrotrophic pathogens in *Arabidopsis*. *Plant Physiol.* 167 1017–1029. 10.1104/pp.114.255505 25593351 PMC4348783

[B71] Nowara D. Gay A. Lacomme C. Shaw J. Ridout C. Douchkov D. (2010). HIGS: host-induced gene silencing in the obligate biotrophic fungal pathogen *Blumeria graminis*. *Plant Cell* 22 3130–3141. 10.1105/tpc.110.077040 20884801 PMC2965548

[B72] Nunes C. C. Dean R. A. (2012). Host-induced gene silencing: a tool for understanding fungal host interaction and for developing novel disease control strategies. *Mol. Plant Pathol.* 13 519–529. 10.1111/j.1364-3703.2011.00766.x 22111693 PMC6638818

[B73] Paparella C. Savatin D. V. Marti L. De Lorenzo G. Ferrari S. (2014). The *Arabidopsis* LYSIN MOTIF-CONTAINING RECEPTOR-LIKE KINASE3 regulates the cross talk between immunity and abscisic acid responses. *Plant Physiol.* 165 262–276. 10.1104/pp.113.233759 24639336 PMC4012585

[B74] Pearce G. Moura D. S. Stratmann J. Ryan C. A. (2001). Production of multiple plant hormones from a single polyprotein precursor. *Nature* 411 817–820. 10.1038/35081107 11459063

[B75] Perchepied L. Balagué C. Riou C. Claudel-Renard C. Rivière N. Grezes-Besset B. (2010). Nitric oxide participates in the complex interplay of defense-related signaling pathways controlling disease resistance to *Sclerotinia sclerotiorum* in *Arabidopsis thaliana*. *Mol. Plant Microbe Interact.* 23 846–860. 10.1094/MPMI-23-7-0846 20521948

[B76] Raffaele S. Farrer R. A. Cano L. M. Studholme D. J. MacLean D. Thines M. (2010). Genome evolution following host jumps in the Irish potato famine pathogen lineage. *Science* 330 1540–1543. 10.1126/science.1193070 21148391

[B77] Raffaele S. Kamoun S. (2012). Genome evolution in filamentous plant pathogens: why bigger can be better. *Nat. Rev. Microbiol.* 10 417–430. 10.1038/nrmicro2790 22565130

[B78] Rietz S. Bernsdorff F. E. Cai D. (2012). Members of the germin-like protein family in *Brassica napus* are candidates for the initiation of an oxidative burst that impedes pathogenesis of *Sclerotinia sclerotiorum*. *J. Exp. Bot.* 63 5507–5519. 10.1093/jxb/ers203 22888126 PMC3444267

[B79] Robert-Seilaniantz A. Grant M. Jones J. D. (2011). Hormone crosstalk in plant disease and defense: more than just jasmonate-salicylate antagonism. *Annu. Rev. Phytopathol.* 49 317–343. 10.1146/annurev-phyto-073009-114447 21663438

[B80] Roux F. Voisin D. Badet T. Balagué C. Barlet X. Huard-Chauveau C. (2014). Resistance to phytopathogens e tutti quanti: placing plant quantitative disease resistance on the map. *Mol. Plant Pathol.* 15 427–432. 10.1111/mpp.12138 24796392 PMC6638617

[B81] Samanta S. Thakur J. K. (2015). Importance of Mediator complex in the regulation and integration of diverse signaling pathways in plants. *Front. Plant Sci.* 6:757 10.3389/fpls.2015.00757 PMC4584954 26442070

[B82] Sanati Nezhad A. Geitmann A. (2013). The cellular mechanics of an invasive lifestyle. *J. Exp. Bot.* 64 4709–4728. 10.1093/jxb/ert254 24014865

[B83] Sanchez-Vallet A. Mesters J. R. Thomma B. P. (2015). The battle for chitin recognition in plant-microbe interactions. *FEMS Microbiol. Rev.* 39 171–183. 10.1093/femsre/fuu003 25725011

[B84] Sarkies P. Miska E. A. (2014). Small RNAs break out: the molecular cell biology of mobile small RNAs. *Nat. Rev. Mol. Cell Biol.* 15 525–535. 10.1038/nrm3840 25053358

[B85] Seifi H. S. Curvers K. De Vleesschauwer D. Delaere I. Aziz A. Hofte M. (2013). Concurrent overactivation of the cytosolic glutamine synthetase and the GABA shunt in the ABA-deficient sitiens mutant of tomato leads to resistance against *Botrytis cinerea*. *New Phytol.* 199 490–504. 10.1111/nph.12283 23627463

[B86] Shlezinger N. Minz A. Gur Y. Hatam I. Dagdas Y. F. Talbot N. J. (2011). Anti-apoptotic machinery protects the necrotrophic fungus *Botrytis cinerea* from host-induced apoptotic-like cell death during plant infection. *PLoS Pathog.* 7:e1002185 10.1371/journal.ppat.1002185 PMC3158046 21876671

[B87] Siewers V. Smedsgaard J. Tudzynski P. (2004). The P450 monooxygenase BcABA1 is essential for abscisic acid biosynthesis in *Botrytis cinerea*. *Appl. Environ. Microbiol.* 70 3868–3876. 10.1128/AEM.70.7.3868-3876.2004 15240257 PMC444755

[B88] Sperschneider J. Dodds P. N. Gardiner D. M. Manners J. M. Singh K. B. Taylor J. M. (2015). Advances and challenges in computational prediction of effectors from plant pathogenic fungi. *PLoS Pathog.* 11:e1004806 10.1371/journal.ppat.1004806 PMC4447458 26020524

[B89] Tariq V. Jeffries P. (1984). Appressorium formation by *Sclerotinia sclerotiorum*: scanning electron microscopy. *Trans. Br. Mycol. Soc.* 82 645–651. 10.1016/S0007-1536(84)80105-9

[B90] Tavormina P. De Coninck B. Nikonorova N. De Smet I. Cammue B. P. (2015). The plant peptidome: an expanding repertoire of structural features and biological functions. *Plant Cell* 27 2095–2118. 10.1105/tpc.15.00440 26276833 PMC4568509

[B91] Wang C. Ding Y. Yao J. Zhang Y. Sun Y. Colee J. (2015a). *Arabidopsis* Elongator subunit 2 positively contributes to resistance to the necrotrophic fungal pathogens *Botrytis cinerea* and *Alternaria brassicicola*. *Plant J.* 83 1019–1033. 10.1111/tpj.12946 26216741

[B92] Wang C. Yao J. Du X. Zhang Y. Sun Y. Rollins J. A. (2015b). The *Arabidopsis* mediator complex subunit16 is a key component of basal resistance against the necrotrophic fungal pathogen *Sclerotinia sclerotiorum*. *Plant Physiol.* 169 856–872. 10.1104/pp.15.00351 26143252 PMC4577384

[B93] Weiberg A. Bellinger M. Jin H. (2015). Conversations between kingdoms: small RNAs. *Curr. Opin. Biotechnol.* 32 207–215. 10.1016/j.copbio.2014.12.025 25622136 PMC4387066

[B94] Weiberg A. Wang M. Bellinger M. Jin H. (2014). Small RNAs: a new paradigm in plant-microbe interactions. *Annu. Rev. Phytopathol.* 52 495–516. 10.1146/annurev-phyto-102313-045933 25090478

[B95] Weiberg A. Wang M. Lin F.-M. Zhao H. Zhang Z. Kaloshian I. (2013). Fungal small RNAs suppress plant immunity by hijacking host RNA interference pathways. *Science* 342 118–123. 10.1126/science.1239705 24092744 PMC4096153

[B96] Williams B. Kabbage M. Kim H. J. Britt R. Dickman M. B. (2011). Tipping the balance: *Sclerotinia sclerotiorum* secreted oxalic acid suppresses host defenses by manipulating the host redox environment. *PLoS Pathog.* 7:e1002107 10.1371/journal.ppat.1002107 PMC3128121 21738471

[B97] Xiao X. Xie J. Cheng J. Li G. Yi X. Jiang D. (2014). Novel secretory protein Ss-Caf1 of the plant-pathogenic fungus *Sclerotinia sclerotiorum* is required for host penetration and normal sclerotial development. *Mol. Plant Microbe Interact.* 27 40–55. 10.1094/MPMI-05-13-0145-R 24299212

[B98] Xu L. Chen W. (2013). Random T-DNA mutagenesis identifies a Cu/Zn superoxide dismutase gene as a virulence factor of *Sclerotinia sclerotiorum*. *Mol. Plant Microbe Interact.* 26 431–441. 10.1094/MPMI-07-12-0177-R 23252459

[B99] Xu L. Xiang M. White D. Chen W. (2015). pH dependency of sclerotial development and pathogenicity revealed by using genetically defined oxalate-minus mutants of *Sclerotinia sclerotiorum*. *Environ. Microbiol.* 17 2896–2909. 10.1111/1462-2920.12818 25720941

[B100] Yin C. Park J. J. Gang D. R. Hulbert S. H. (2014). Characterization of a tryptophan 2-monooxygenase gene from *Puccinia graminis* f. sp. tritici involved in auxin biosynthesis and rust pathogenicity. *Mol. Plant Microbe Interact.* 27 227–235. 10.1094/MPMI-09-13-0289-FI 24350783

[B101] Zhang H. Wu Q. Cao S. Zhao T. Chen L. Zhuang P. (2014). A novel protein elicitor (SsCut) from *Sclerotinia sclerotiorum* induces multiple defense responses in plants. *Plant Mol. Biol.* 86 495–511. 10.1007/s11103-014-0244-3 25149470

[B102] Zhang L. Kars I. Essenstam B. Liebrand T. W. Wagemakers L. Elberse J. (2014). Fungal endopolygalacturonases are recognized as microbe-associated molecular patterns by the *Arabidopsis* receptor-like protein RESPONSIVENESS TO BOTRYTIS POLYGALACTURONASES1. *Plant Physiol.* 164 352–364. 10.1104/pp.113.230698 24259685 PMC3875813

[B103] Zhang W. Fraiture M. Kolb D. Loffelhardt B. Desaki Y. Boutrot F. F. (2013). *Arabidopsis* receptor-like protein30 and receptor-like kinase suppressor of BIR1-1/EVERSHED mediate innate immunity to necrotrophic fungi. *Plant Cell* 25 4227–4241. 10.1105/tpc.113.117010 24104566 PMC3877809

[B104] Zhang X. Wang C. Zhang Y. Sun Y. Mou Z. (2012). The *Arabidopsis* mediator complex subunit16 positively regulates salicylate-mediated systemic acquired resistance and jasmonate/ethylene-induced defense pathways. *Plant Cell* 24 4294–4309. 10.1105/tpc.112.103317 23064320 PMC3517251

[B105] Zhang Y. Li D. Zhang H. Hong Y. Huang L. Liu S. (2015). Tomato histone H2B monoubiquitination enzymes SlHUB1 and SlHUB2 contribute to disease resistance against *Botrytis cinerea* through modulating the balance between SA- and JA/ET-mediated signaling pathways. *BMC Plant Biol.* 15:252 10.1186/s12870-015-0614-2 PMC4618151 26490733

[B106] Zhao X. Han Y. Li Y. Liu D. Sun M. Zhao Y. (2015). Loci and candidate gene identification for resistance to *Sclerotinia sclerotiorum* in soybean (*Glycine max* L. Merr.) via association and linkage maps. *Plant J.* 82 245–255. 10.1111/tpj.12810 25736370

[B107] Zhou J. Fu Y. Xie J. Li B. Jiang D. Li G. (2012). Identification of microRNA-like RNAs in a plant pathogenic fungus *Sclerotinia sclerotiorum* by high-throughput sequencing. *Mol. Genet. Genomics* 287 275–282. 10.1007/s00438-012-0678-8 22314800

[B108] Zhou J. Zeng L. Liu J. Xing D. (2015). Manipulation of the xanthophyll cycle increases plant susceptibility to *Sclerotinia sclerotiorum*. *PLoS Pathog.* 11:e1004878 10.1371/journal.ppat.1004878 PMC4439079 25993128

[B109] Zhu W. Wei W. Fu Y. Cheng J. Xie J. Li G. (2013). A secretory protein of necrotrophic fungus *Sclerotinia sclerotiorum* that suppresses host resistance. *PLoS ONE* 8:e53901 10.1371/journal.pone.0053901 PMC3544710 23342034

[B110] Zhu Y. Schluttenhoffer C. M. Wang P. Fu F. Thimmapuram J. Zhu J. K. (2014). CYCLIN-DEPENDENT KINASE8 differentially regulates plant immunity to fungal pathogens through kinase-dependent and -independent functions in *Arabidopsis*. *Plant Cell* 26 4149–4170. 10.1105/tpc.114.128611 25281690 PMC4247566

